# Evaluating the perspectives of ChatGPT and Gemini on glenohumeral osteoarthritis management

**DOI:** 10.1016/j.jseint.2025.03.011

**Published:** 2025-04-10

**Authors:** Michael Megafu, Omar Guerrero, Rafay Hasan, Larry Hunt, Devri Langhelm, Benning Le, Xinning Li, Robert Kelly, Robert L. Parisien, Antonio Cusano

**Affiliations:** a Department of Orthopaedic Surgery, University of Connecticut, Farmington, CT, USA; b A.T. Still University School of Osteopathic Medicine in Arizona, Mesa, AZ, USA; c Department of Orthopedic Surgery, Boston University School of Medicine, Boston, MA, USA; d Department of Orthopedic Surgery, University of Pennsylvania, Philadelphia, PA, USA; e Department of Orthopedic Surgery, Mount Sinai, New York, NY, USA

**Keywords:** Artificial intelligence, AI, ChatGPT, Gemini, Glenohumeral arthritis, AAOS CPG

## Abstract

**Background:**

Integrating machine learning and artificial intelligence (AI) technologies has revolutionized various sectors, including health care. However, their application in orthopedic health-care settings still needs to be improved. This study sought to evaluate Chat Generative Pre-Trained Transformer (ChatGPT) and Gemini's capacity to make quality medical recommendations regarding glenohumeral osteoarthritis, weighing them against the recommendations established in the Evidence-Based Clinical Practice Guidelines (CPGs) of the American Academy of Orthopaedic Surgeons (AAOS).

**Methods:**

The 2020 AAOS CPGs, a widely recognized and respected source, were the basis for determining recommended and nonrecommended treatments in this study. ChatGPT and Gemini were queried on 20 treatments based on these guidelines; 10 were recommended for managing glenohumeral joint osteoarthritis, five were not recommended for managing glenohumeral joint osteoarthritis, and five were reported as consensus statements. These responses were categorized as “Concordance” or “No Concordance” with the AAOS CPGs. A Cohen's Kappa coefficient was calculated to assess the interrater reliability.

**Results:**

Among the 20 treatments examined, ChatGPT and Gemini showed concordance with the AAOS CPGs for 10 (100%) and 5 (50%) treatments, respectively. On the other hand, for treatments that AAOS CPGs did not recommend, ChatGPT had concordance for four out of the five treatments (80%), while Gemini had 100% concordance. The Cohen's Kappa coefficient to assess interrater reliability was found to be 0.90, indicating a very high level of agreement between the two raters in categorizing responses as “Concordance” or “No Concordance” with the AAOS CPGs.

**Conclusion:**

The study findings reveal that ChatGPT and Gemini cannot solely recommend CPGs as outlined in AAOS CPGs. As patients increasingly utilize external resources such as AI platforms and the Internet for medical recommendations, providers should advise patients to exercise caution when seeking medical advice from these AI platforms for managing glenohumeral joint osteoarthritis.

Orthopedic surgery has begun to integrate machine learning (ML) and artificial intelligence (AI) to help aid in better outcomes when administering health care.^5^ Many large language models (LLMs) have been utilized in specific applications, such as supplementing radiology-based diagnostics in low-resource emergency departments.^9^ In orthopedics, there have been strides in AI to help aid patients. Yet, an understanding of effectively utilizing AI while addressing ethical concerns related to bias, privacy, and accountability remains to be realized.^3^ Despite the evidence-based practices of modern medicine, patients utilize other means for obtaining information. Patients must be educated to ensure that informed consent, patient autonomy, and patient satisfaction are optimized to provide the best patient outcomes.^20^ Many patients use the Internet and social media to research their injuries and seek advice on treatment plans.^1^^,^^4^ The medical information on the Internet needs to be vetted by health-care professionals and can contain false information, leading to misinformed patients and retrograded patient education.^14^

The advancement of AI-powered chatbots, the Internet, social media platforms, AI, and other LLM have given rise to ongoing searches for patients to build their knowledge bases. These chatbots include Chat Generative Pre-Trained Transformer (ChatGPT; OpenAI, San Francisco, CA, USA), and Gemini (previously known as Bard, created by Google; Gemini; Alphabet Inc., Mountain View, CA, USA), and they have an increasing capacity to communicate complex prompts and queries.^16^ As the responses of these chatbots become progressive, human-like, and relatable, their ability to produce digestible information to consumers makes them highly functional for educating people.^6^ Patients with specific injuries consult these LLMs to improve their knowledge to justify their decision to consult a doctor. ChatGPT developers are trained to receive information from Wikipedia and other publicly available resources as their source of knowledge, which threatens the validity of their recommendations and makes them susceptible to the reiteration of false information.^7^^,^^8^

Glenohumeral osteoarthritis (GHOA) is the third most common form of osteoarthritis after hip and knee osteoarthritis.^10^ GHOA is characterized by the degeneration of articular cartilage of the humeral head and can occur as a primary (degeneration of cartilage over time) or secondary (trauma, shoulder dislocation, shoulder instability, massive rotator cuff tears, and inflammatory arthropathy) cause.^10^ There has been controversy regarding the optimal management of GHOA, and evaluating the current management of this pathology is essential to providing evidence-based treatment. The purpose of this study is to evaluate ChatGPT and Gemini's capacity to make quality medical recommendations regarding GHOA and compare them against the recommendations established in the evidence-based Clinical Practice Guidelines (CPGs) of the American Academy of Orthopaedic Surgeons (AAOS). We hypothesize that ChatGPT and Gemini will not provide accurate recommendations for every treatment based on the AAOS CPGs.

## Methods

The CPGs of the AAOS are expert-developed, evidence-based recommendations to guide health-care providers in making informed decisions regarding patient care in orthopedic surgery. These guidelines encompass suggestions for diagnosis, treatment, and post-treatment care, drawing from the latest research and expert consensus through evidence-based medicine. These recommendations are graded based on their strength, utilizing a classification system that includes categories such as “Strong,” “Moderate,” “Limited,” and “Consensus.” The “Consensus” category indicates recommendations based on expert judgment when insufficient or conflicting evidence is available. The 2020 AAOS CPGs on managing GHOA served as the basis for determining recommended and nonrecommended treatments in this study.

The research team outlined 20 recommendations for managing GHOA, dividing them into ten treatment recommendations (Table I) and ten recommendations against treatment (Table II). To assess the efficacy of these treatments, impartial inquiries were made to ChatGPT (version 4; ChatGPT-4) and Gemini, ensuring unbiased evaluations. All 20 treatments were queried on the same day to avoid temporal biases. The responses were categorized as “Concordance” or “No Concordance” based on their alignment with the AAOS CPGs recommendations. The agreement between raters was measured using Cohen's Kappa coefficient to evaluate interrater reliability between two raters.

**Table I:** Recommended glenohumeral interventions queried.

Topic	American Academy of Orthopaedic Surgeons (AAOS) recommendation	Strength of recommendation	Query
Total shoulder arthroplasty	Strong evidence supports that anatomic total shoulder arthroplasty demonstrates more favorable function and pain relief in the short—to mid-term follow-up than hemiarthroplasty for the treatment of glenohumeral osteoarthritis.	Strong	Is total shoulder arthroplasty recommended over hemiarthroplasty for treating glenohumeral joint osteoarthritis?
Glenoid components (pegged or keeled)	Strong evidence supports the clinician's use of pegged or keeled glenoid components in patients with glenohumeral joint osteoarthritis and a well-functioning rotator cuff. Pegged components demonstrate fewer radiolucent lines, but the effect on clinical outcomes and survivorship is unclear.	Strong	Are pegged or keeled glenoid components recommended for treating glenohumeral joint osteoarthritis?
Total shoulder arthroplasty -- subscapularis peel	Moderate evidence supports surgeons utilizing subscapularis peel, lesser tuberosity osteotomy, or tenotomy when performing shoulder arthroplasty.	Moderate	Is a subscapularis peel recommended when performing shoulder arthroplasty?
Total shoulder arthroplasty -- tenotomy	Moderate evidence supports that surgeons can utilize tenotomy when performing shoulder arthroplasty.	Moderate	Is tenotomy recommended when performing shoulder arthroplasty?
Total shoulder arthroplasty -- lesser tuberosity osteotomy	Moderate evidence supports that surgeons can utilize lesser tuberosity osteotomy when performing shoulder arthroplasty.	Moderate	Is lesser tuberosity osteotomy recommended when performing shoulder arthroplasty?
Hemiarthroplasty – stems	Limited evidence supports that clinicians may utilize stemmed prostheses for patients with glenohumeral joint osteoarthritis undergoing total or hemiarthroplasty.	Limited	Are stemmed, stemless, or resurfacing prostheses recommended for patients with glenohumeral joint osteoarthritis undergoing total or hemiarthroplasty?
Cemented stems	In the absence of reliable evidence, the work group believes that either cemented or cementless stems can be utilized in the treatment of patients with glenohumeral joint osteoarthritis and a well-functioning rotator cuff.	Consensus	Are cemented or cementless stems recommended for treating a well-functioning rotator cuff in a patient with glenohumeral joint osteoarthritis?
Glenoid components - polyethylene metal or all polyethylene	In the absence of reliable evidence, it is the opinion of the workgroup that clinicians may use polyethylene metal hybrid glenoid components or all polyethylene components during total shoulder arthroplasty for the treatment of glenohumeral joint osteoarthritis	Consensus	Are polyethylene-metal hybrid glenoid or all-polyethylene components recommended for glenoid components during a total shoulder arthroplasty to treat glenohumeral joint osteoarthritis?
Biceps tenodesis and tenotomy	In the absence of reliable evidence, the workgroup believes clinicians may consider concomitant biceps tenodesis or tenotomy during shoulder arthroplasty.	Consensus	Is a biceps tenodesis or tenotomy recommended during a shoulder arthroplasty?
Cryotherapy	In the absence of reliable evidence, the workgroup believes that either continuous cryotherapy or cold packs can be used following shoulder arthroplasty.	Consensus	Is continuous cryotherapy or cold packs recommended for use following shoulder arthroplasty?

**Table II:** Glenohumeral interventions recommended against queried.

Topic	American Academy of Orthopaedic Surgeons (AAOS) recommendation	Strength of recommendation	Query
Hyaluronic acid	Strong evidence supports that there is no benefit to using hyaluronic acid in treating glenohumeral joint osteoarthritis.	Strong	Is hyaluronic acid recommended in the presence of glenohumeral joint osteoarthritis?
Glenoid components – metal-backed cementless	Moderate evidence supports that surgeons should not use metal-backed cementless glenoid components.	Moderate	Are metal-backed cementless glenoid components recommended in a total shoulder arthroplasty?
Opioid pain medication	In the absence of reliable evidence, it is the opinion of the workgroup that opioids should not be prescribed as routine and long-term pain management of glenohumeral osteoarthritis.	Consensus	Are opioids prescribed for the management of glenohumeral osteoarthritis?
Injectable biologics - stem cells	The work group believes that injectable biologics, such as stem cells or platelet-rich plasma, cannot be recommended for the treatment of glenohumeral osteoarthritis in the absence of reliable evidence.	Consensus	Are injectable stem cells recommended for the treatment of glenohumeral osteoarthritis?
Injectable biologics - platelet-rich plasma	The work group believes that injectable biologics involving stem cells cannot be recommended for the treatment of glenohumeral osteoarthritis in the absence of reliable evidence.	Consensus	Are injectable platelet-rich plasma recommended for the treatment of glenohumeral osteoarthritis?
Not recommended for or against by AAOS CPGs			
Alternative nonsurgical treatments	In the absence of reliable evidence, the work group cannot recommend acupuncture for or against use.	Consensus	Is acupuncture recommended for the treatment of glenohumeral osteoarthritis?
Alternative nonsurgical treatments	The work group cannot recommend or against dry needling without reliable evidence.	Consensus	Is dry needling recommended for the treatment of glenohumeral osteoarthritis?
Alternative nonsurgical treatments	Without reliable evidence, the workgroup cannot recommend or be against using glucosamine and chondroitin.	Consensus	Are glucosamine and chondroitin recommended for the treatment of glenohumeral osteoarthritis?
Alternative nonsurgical treatments	In the absence of reliable evidence, the workgroup cannot recommend whether to use cupping.	Consensus	Is cupping recommended for the treatment of glenohumeral osteoarthritis?
Alternative nonsurgical treatments	Without reliable evidence, the work group cannot recommend or against using TENS.	Consensus	Is TENS recommended for the treatment of glenohumeral osteoarthritis?

*CPG*, clinical practice guidelines; *TENS*, transcutaneous electrical nerve stimulation.

The institutional review board was not necessary for this study as this study did not involve human subjects.

## Results

Among the 20 treatments examined, both ChatGPT-4 and Gemini showed concordance with the AAOS CPGs for 10 (100%) and 5 (50%) treatments, respectively (Table III). Table IV illustrates the concordance of both ChatGPT-4 and Gemini with the AAOS CPGs recommendations. ChatGPT-4 exhibited concordance with all the recommended treatments by the AAOS CPGs (100%), while Gemini showed concordance for only five out of the ten treatments (50%). In contrast, ChatGPT-4 had concordance for treatments not recommended by the AAOS CPGs, with four out of the five treatments (80%), while Gemini showed 100% concordance. Regarding treatments not explicitly recommended for or against the AAOS CPGs, ChatGPT-4 achieved 100% concordance with the five mentioned options, whereas Gemini produced concordance for only 40% of these treatments. The Cohen's Kappa coefficient, used to assess interrater reliability, was found to be 0.90, indicating a very high level of agreement between the two raters in categorizing responses as “Concordance” or “No Concordance” with the AAOS CPGs. Table V shows nonconcordant responses regarding the CPGs by AAOS recommendations.

**Table III:** ChatGPT and Gemini responses concordance with AAOS clinical practice guidelines by AAOS recommendations.

Recommendations (N = 20)	ChatGPT		Gemini		Concordance rate	Recommendations (N = 20)
	Concordance n (%)	No Concordance n (%)	Concordance n (%)	No Concordance n (%)		
AAOS - recommended (10)	10 (100%)	0	5 (50%)	5 (50%)	15/20 (75%)	AAOS - Recommended (10)
AAOS - not recommended (5)	4 (80%)	1 (20%)	5 (100%)	0	9/10 (90%)	AAOS - Not Recommended (5)
AAOS -neutral (5)	5 (100%)	0	2 (40%)	3 (60%)	7/10 (70%)	AAOS -Neutral (5)

*ChatGPT*, Chat Generative Pretrained Transformer; *AAOS*, American Academy of Orthopaedic Surgeons.

**Table IV:** ChatGPT and Gemini responses concordance with AAOS clinical practice guidelines by AAOS recommendations.

Recommendations	Recommended by AAOS CPG	ChatGPT responses: Concordance with AAOS CPG	Gemini responses: Concordance with AAOS CPG
Recommended by AAOS CPG			
Total shoulder arthroplasty	Yes	Yes	Yes
Glenoid components (pegged or keeled)	Yes	Yes	No
Total shoulder arthroplasty -- subscapularis peel	Yes	Yes	No
Total shoulder arthroplasty -- tenotomy	Yes	Yes	No
Total shoulder arthroplasty -- lesser tuberosity osteotomy	Yes	Yes	No
Hemiarthroplasty – stems	Yes	Yes	Yes
Cemented stems	Yes	Yes	Yes
Glenoid components - polyethylene metal or all polyethylene	Yes	Yes	Yes
Biceps tenodesis and tenotomy	Yes	Yes	No
Cryotherapy	Yes	Yes	Yes
Not recommended by AAOS CPG			
Hyaluronic acid	No	No	Yes
Glenoid components – metal-backed cementless	No	Yes	Yes
Opioid pain medication	No	Yes	Yes
Injectable biologics - stem cells	No	Yes	Yes
Injectable biologics - platelet-rich plasma	No	Yes	Yes
Not recommended for or against by AAOS CPG			
Alternative nonsurgical treatments - acupuncture	Neutral	Yes	No
Alternative nonsurgical treatments – dry needling	Neutral	Yes	Yes
Alternative nonsurgical treatments - glucosamine and chondroitin	Neutral	Yes	No
Alternative nonsurgical treatments - cupping	Neutral	Yes	Yes
Alternative nonsurgical treatments - TENS	Neutral	Yes	No

*ChatGPT*, Chat Generative Pretrained Transformer; *AAOS*, American Academy of Orthopaedic Surgeons; *CPG*, clinical practice guidelines; *TENS*, transcutaneous electrical nerve stimulation.

**Table V:** Nonconcordant ChatGPT and Gemini responses regarding the clinical practice guidelines by AAOS recommendations.

Query	ChatGPT response:	Gemini response:
Recommended by AAOS CPG		
Strong evidence supports the clinician's use of pegged or keeled glenoid components in patients with glenohumeral joint osteoarthritis and a well-functioning rotator cuff. Pegged components demonstrate fewer radiolucent lines, but the effect on clinical outcomes and survivorship is unclear.		“Pegged or keeled glenoid components aren't universally recommended for osteoarthritis.”
Moderate evidence supports surgeons utilizing subscapularis peel, lesser tuberosity osteotomy, or tenotomy when performing shoulder arthroplasty.		“A subscapularis peel is not necessarily recommended for shoulder arthroplasty.”
Moderate evidence supports that surgeons can utilize tenotomy when performing shoulder arthroplasty.		“Tenotomy is possible during shoulder arthroplasty, but it depends on the specific tendon and the situation. Tenotomy is a last resort during shoulder arthroplasty.”
Moderate evidence supports that surgeons can utilize lesser tuberosity osteotomy when performing shoulder arthroplasty.		“LTO can be a valuable technique for some shoulder arthroplasty patients, but it's not universally recommended.”
In the absence of reliable evidence, the workgroup believes clinicians may consider concomitant biceps tenodesis or tenotomy during shoulder arthroplasty.		“Biceps tenotomy is not a recommended course of action during shoulder arthroplasty. Biceps tenodesis is possible, but only if the biceps tendon is causing issues.”
Not recommended by AAOS CPG		
Strong evidence supports that there is no benefit to using hyaluronic acid in treating glenohumeral joint osteoarthritis.	“Hyaluronic acid injections are sometimes recommended as a treatment option for glenohumeral joint osteoarthritis. These injections can help alleviate pain and improve joint lubrication for some patients, although the effectiveness can vary from person to person.”	
Neutral recommendation by AAOS CPG		
The workgroup cannot recommend acupuncture for or against it without reliable evidence.		“Acupuncture is not definitively recommended for the treatment of glenohumeral osteoarthritis.”
Without reliable evidence, the workgroup cannot recommend for or against using glucosamine and chondroitin.		“Glucosamine and chondroitin are not recommended for treating glenohumeral osteoarthritis.”
Without reliable evidence, the work group cannot recommend for or against using TENS.		“TENS is not recommended for the treatment of glenohumeral osteoarthritis. Here's the breakdown.”

*ChatGPT*, Chat Generative Pretrained Transformer; *AAOS*, American Academy of Orthopaedic Surgeons; *CPG*, clinical practical guidelines; *LTO*, lesser tuberosity osteotomy; *TENS*, transcutaneous electrical nerve stimulation.

## Discussion

Using language learning models such as ChatGPT and Gemini in health care has sparked considerable interest due to their potential to provide accessible and personalized information to patients and clinicians.^12^^,^^18^ However, this study assessed the validity of ChatGPT and Gemini when queried to recommend or not recommend treatments of GHOA. This study found that ChatGPT was in concordance with AAOS CPGs on every queried medical treatment, except for a recommendation against using hyaluronic acid for GHOA pain management. ChatGPT reached 100% concordance on treatments that were either recommended or neutrally by the AAOS CPGs (Table III). Gemini showed 50% concordance (50% no concordance) with AAOS CPGs on recommended treatments but 100% concordance on nonrecommended therapies and 40% concordance (60% no concordance). The AAOS CPGs held neutral views. Thus, our hypothesis was correct, as ChatGPT and Gemini failed to respond accurately to every treatment recommendation.

Several recent studies have examined the possible application of LLMs in health care and raised doubts about their capacity to generate responses consistent with medical advice.^12^, ^13^, ^14^, ^15^, ^16^ Vaid et al discovered that Chat GPT-4 could autonomously operate within a clinical environment, effectively ordering relevant tests and adhering to clinical protocols.^18^ Armitage evaluated Chat GPT-4 using four publicly available sample papers for the Membership of the Royal College of Pediatrics and Child Health theory exams, achieving an average score of 78% across all papers.^2^ Walker et al investigated ChatGPT's responses to queries about five common hepatopancreatic biliary conditions, revealing a concordance rate of only 60% with United Kingdom National Institute for Health and Care Excellence guidelines.^19^ Rahsepar et al compared ChatGPT, Gemini, and non-AI search engines on questions related to lung cancer prevention, screening, and terminology, finding that all platforms had similar performance, with accuracy levels ranging from 55% to 70%.^11^

Studies examining LLMs in health care consistently highlight their potential and limitations. While LLMs like Chat GPT-4 and others have shown promise in providing accurate information, they have yet to achieve 100% accuracy. This is particularly critical in medical information, where precision and reliability are paramount for patient safety and effective health-care delivery. The findings from these studies align with our observations, such as the concordance rates observed with ChatGPT and Gemini concerning the AAOS glenohumeral joint CPGs. These results underscore the capability of LLMs to align with established clinical guidelines and protocols to a significant extent. However, the gap between 100% accuracy highlights the ongoing challenge of ensuring that AI-driven systems consistently provide medically sound and evidence-based information. One concerning aspect highlighted by these studies is the high concordance rates observed with limited and consensus nonrecommended treatments. This raises questions about the sources from which LLMs obtain information regarding less conventional or nonstandard medical practices. It also underscores the imperative for ongoing evaluation, validation, and enhancement of AI models within health-care settings.

One significant drawback of LLMs in the health-care domain is their reliance on training data derived from vast text collections, including sources like the Internet, Wikipedia, books, newspapers, and various documents.^13^ While this provides LLMs with a broad understanding of medical concepts, it poses challenges, particularly in specialized areas like orthopedics. Despite having access to specialized medical literature and research, LLMs often require specific programming or feedback mechanisms to refine their training toward higher-quality textual sources.^15^^,^^17^ This limitation arises because LLMs' general training data may only sometimes reflect the depth and nuance required for complex medical domains like orthopedics. These models must learn to distinguish between reliable medical information and potentially misleading content in broader text corpora.

This study had potential limitations. First, the classification of these responses is subjective, which could introduce bias. However, the two reviewers searched simultaneously, and a Cohen's Kappa coefficient of 0.90 demonstrates a near-perfect interrater reliability. The answers may change among different language model versions with the multiple updates and versions of ChatGPT and Gemini. Thus, the information and answers from Gemini and ChatGPT may be based on the 2017 AAOS CPGs guidelines instead of the newer 2020 guidelines. Perhaps future studies can outline the differences between the guidelines and examine the concordance rate of ChatGPT, Gemini, and other LLMs. While the CPG recommendations from the AAOS were classified as concordance, discordance, or no concordance, we only analyzed a subset of AAOS CPG topics, which can introduce selection bias. Lastly, the questions in this study may not necessarily contextualize the full spectrum of medical questions about managing GHOA, which may limit our results.

## Conclusion

The study findings reveal that ChatGPT and Gemini cannot be solely used to recommend CPGs as outlined in AAOS CPGs. As patients increasingly utilize external resources such as AI platforms and the Internet for medical recommendations, providers should advise patients to exercise caution when seeking medical advice from these AI platforms for managing glenohumeral joint osteoarthritis.

## Disclaimers

Funding: No funding was disclosed by the authors.

Conflicts of interest: Robert L Parisien reports the following potential conflicts: has received education payments from Gotham Surgical and Arthrex and a grant from Arthrex. All the other authors, their immediate families, and any research foundation with which they are affiliated have not received any financial payments or other benefits from any commercial entity related to the subject of this article.
